# Be aware of the “O” sign in the bipolar cup dissociation during closed reduction of bipolar dislocation: A case report

**DOI:** 10.1097/MD.0000000000035234

**Published:** 2023-09-15

**Authors:** Seung-Bo Sim, Sang-Woo Son, Bum-Jin Shim

**Affiliations:** a Department of Orthopedic Surgery, Kyungpook National University Hospital, Jung-gu, Daegu, South Korea; b Department of Orthopaedic Surgery, Kyungpook National University, College of Medicine, Jung-gu, Daegu, South Korea; c Department of Orthopedic Surgery, Kyungpook National University Chilgok Hospital, Buk-gu, Daegu, South Korea.

**Keywords:** bipolar, dislocation, dissociation, hemiarthroplasty, locking

## Abstract

**Rationale:**

Bipolar cup dissociation following hip hemiarthroplasty is a rare complication of which only a few cases have been reported, and it usually requires revision surgery because of difficulties in closed reduction.

**Patient concerns:**

We report the case of a 57-year-old man who underwent bipolar hemiarthroplasty 2 months ago caused by a left femoral neck fracture. Postoperatively, bipolar dislocation occurred thrice, and the patient showed bipolar cup dissociation during closed reduction maneuver at the recent visit. At the time of this event, no consideration was given to the shape of the prosthesis on the radiograph.

**Diagnoses:**

The patient was diagnosed with early bipolar cup dissociation.

**Interventions:**

The patient underwent revision surgery to replace and reassemble the femoral head component.

**Outcomes:**

No further dislocation occurred following the surgery.

**Lessons:**

To avoid dissociation of the components during closed reduction, it would be helpful to have knowledge of the “O” sign, a concentric circle shape of the prosthesis on the radiograph.

**Level of evidence:**

Level V, case report.

## 1. Introduction

Bipolar cup dissociation is a rare but potentially serious complication following hip hemiarthroplasty.^[1,2]^ Although the articulation of bipolar cup and femoral head component is locked and strong, bipolar cup dissociation may occur, which requires revision surgery because closed reduction is impossible.^[3]^ Unlike late bipolar cup dissociation, which occurs following wear and breakage of internal structures, such as the locking ring or liner, early bipolar cup dissociation occurs during closed reduction maneuver.^[4]^

Herein, we present a case of early bipolar cup dissociation and aim to increase awareness for this rare but important complication of hip hemiarthroplasty to provide guidance on its management, including radiographic consideration.

## 2. Case report

**Figure 1.**
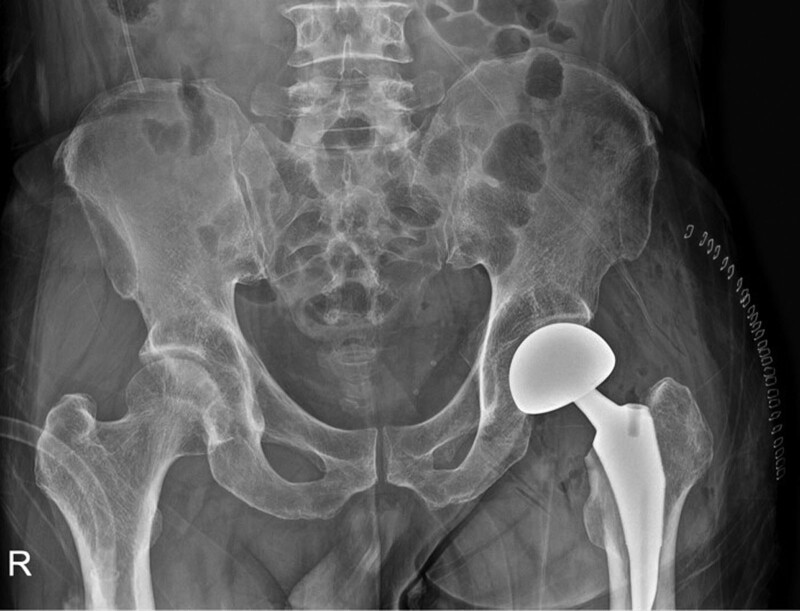
Patient initial postoperative anteroposterior radiograph of hip hemiarthroplasty.

A 57-year-old man came to emergency department of our hospital complaining of left hip pain with a history of slip-down. He sustained a displaced femoral neck fracture. He had left-sided hemiplegia with a history of craniotomy and tumor removal for glioblastoma in 2020. Furthermore, re-exploration with craniotomy was performed for a brain tumor recurrence in 2021. Subsequently, he had a limitation of left-side weight-bearing with a muscle grade of 3. Given that life expectancy is <1 year, the patient was treated using cementless bipolar hemiarthroplasty through the posterolateral approach (Fig. 1). The RINGLOC Cup (Biomet, Warsaw, IN) with a dual locking mechanism was combined with a TaperLoc cementless stem (Biomet, Warsaw, IN). The cup size was 51 mm, and it had a 28-mm BIOLOX Delta (CeramTec, Plochingen, Germany) alumina ceramic head.

**Figure 2.**
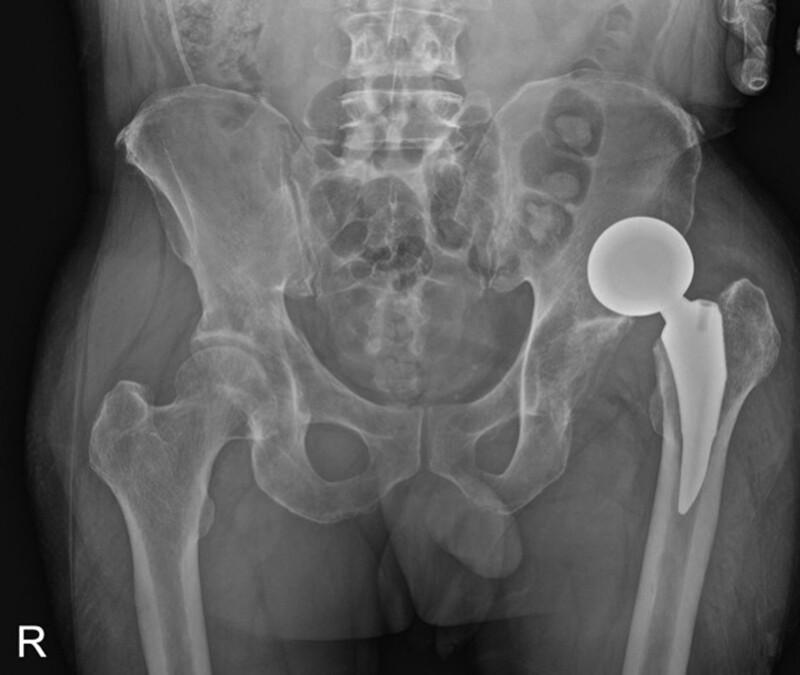
Anteroposterior radiograph demonstrating the first dislocation in hip hemiarthroplasty. The femoral head component appeared as an “O”-shaped concentric circle.

**Figure 3.**
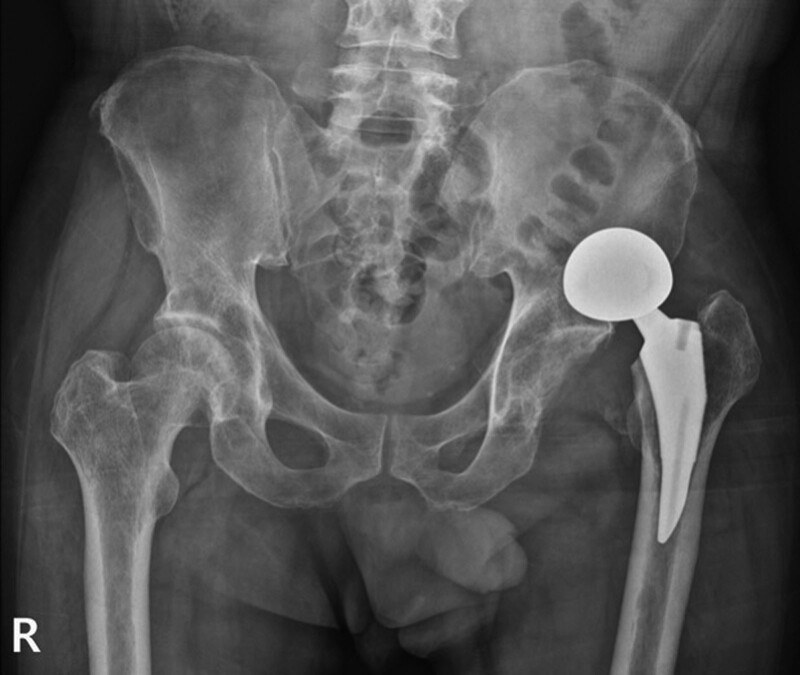
Anteroposterior radiograph demonstrating the third dislocation in hip hemiarthroplasty. The femoral head component appeared as an elliptical shape and not “O”-shaped.

**Figure 4.**
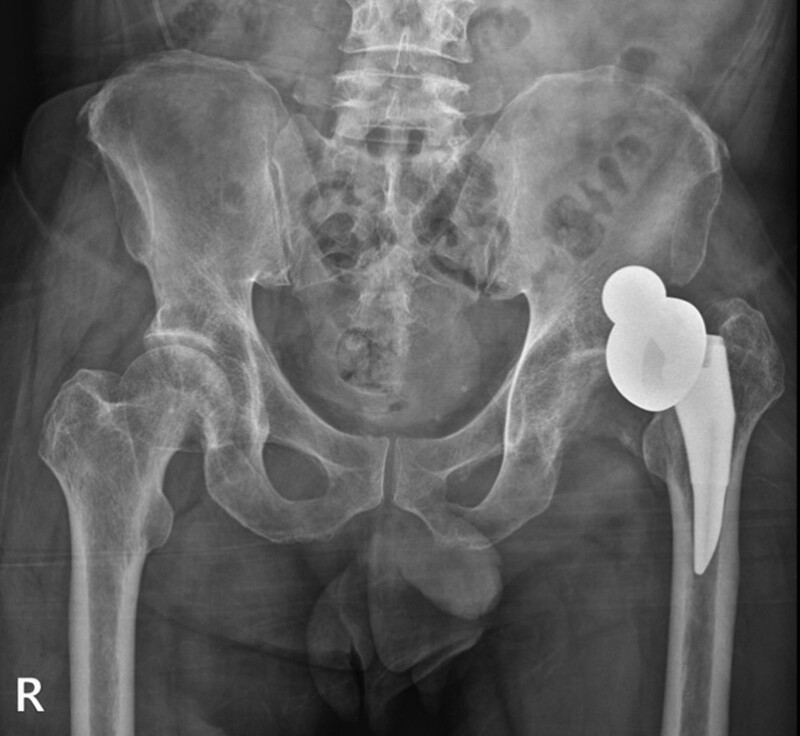
Anteroposterior radiograph showing the dissociation between bipolar cup components following manual reduction.

**Figure 5.**
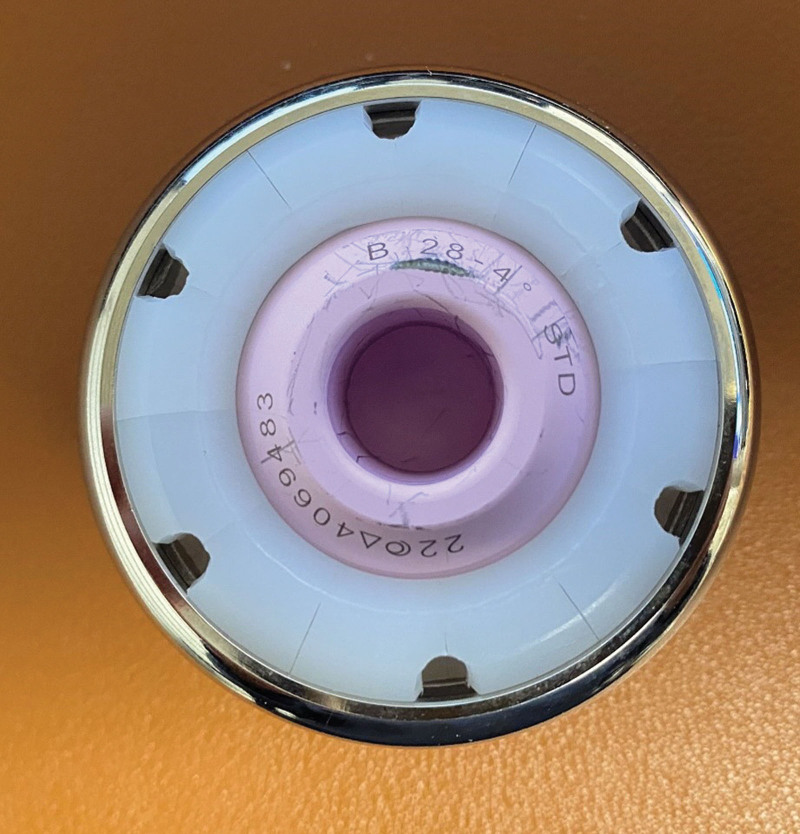
Photograph of the combined bipolar head component (RINGLOC Cup and BIOLOX Delta). It was not worn out, or the locking mechanism was broken.

Eight weeks later, while getting out of bed, he sustained posterior dislocation of the prosthesis, and the femoral head component appeared with an “O” shape, concentric circle on the anteroposterior (AP) radiograph (Fig. 2), and manual reduction was performed successfully. After 2 days, dislocation recurred, and manual reduction was performed again. At this time, a hip brace was applied to maintain the posture and prevent dislocation. However, 3 weeks later, posterior dislocation occurred again (Fig. 3). An AP radiograph showed an elliptical femoral head component. During manual reduction, bipolar cup dissociation occurred (Fig. 4). Open reduction was undertaken, and intraoperatively, no signs of severe wear of the prosthesis were observed; when re-assembled, it showed strong bonding, and the locking mechanism was intact (Fig. 5). Given the slightly lax left hip and muscle weakness, we changed 28-mm BIOLOX Delta into 28 mm + 3 BIOLOX Delta, and the prosthesis was confirmed to be stable throughout its full range of motion. Postoperatively, the patient demonstrated a drowsy mentality, and brain computed tomography confirmed the enlargement of the brain tumor. No further dislocation occurred 1 month following the surgery; however, the patient died because of underlying disease in the second month. The written informed consent was obtained from his wife.

## 3. Discussion

Dislocation in hip hemiarthroplasty is uncommon, and the overall rate reported was 3.4%.^[5]^ Bipolar cup dissociation is a rare complication, and only a few case reports have been published.^[1–3,6–8]^ Although the patient characteristics and instruments used are different, an inherent factor of the modular system of the bipolar cup and femoral head is a possibility of bipolar cup dissociation.

Bipolar cup dissociation is divided into early and late dissociation, and the mechanism by which it occurs varies.^[4,9]^ The eccentric wear of the polyethylene liner is the primary cause of late dissociation and breakage of the locking ring between the polyethylene liner and the femoral head. On the contrary, as shown in our case, early dissociation can occur during manual reduction of a dislocated hip hemiarthroplasty. Referred to as the “bottle-opener effect,” and this dissociation is caused by the bipolar cup being encroached the posterior acetabular rim during the closed reduction.^[10]^

**Figure 6.**
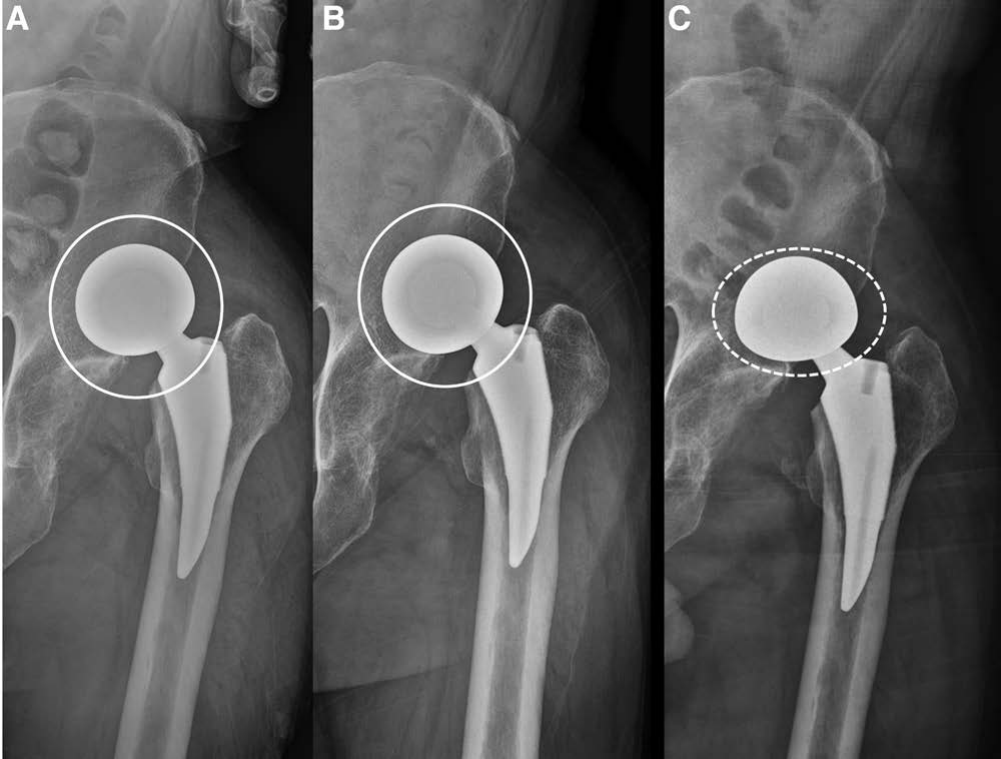
Anteroposterior radiograph demonstrating dislocation in hip hemiarthroplasty. (A) The first dislocation occurred 8 wk following surgery, and the femoral head component appeared as an “O”-shaped concentric circle. (B) The second dislocation occurred 2 d after the first dislocation. The femoral head component appeared as an “O”-shaped concentric circle. (C) The third dislocation occurred. The femoral head component appeared elliptical.

In the current case, a total of 3 dislocations occurred, and the radiographic difference was found in the closed reduction maneuver in which dissociation occurred (Fig. 6). Reduction was successful in the case of an “O”-shaped concentric circle cup and head on AP radiograph; however, the dissociation occurred while attempting reduction in the case of an elliptical femoral head component. In this case, it is believed that before the reduction of dislocation of hip hemiarthroplasty, if we carefully focus on the radiographic shape of the prosthesis, the possibility of bipolar cup dissociation may occur. If an “O” shape is not observed on the AP radiograph, reduction should be attempted while taking a dynamic fluoroscopic image with sufficient patient sedation and avoiding the application of a twisting force. In addition, we suggest naming “O” sign as “O”-shape on the AP radiograph to emphasize the importance of this shape and avoid or minimize the bipolar cup dissociation during closed reduction of bipolar dislocation.

Previous report of Lee et al showed that a single locking mechanism rather than a dual locking mechanism may be a risk factor for bipolar dissociation.^[9]^ In their report, only 1 of 7 bipolar cup dissociation cases had a dual locking mechanism. In the present study, we used the RINGLOC Cup with a dual locking mechanism. Although the dual locking mechanism is more stable than the single locking mechanism, care must be taken to avoid this rare complication.

Undoubtedly, the management of bipolar cup dissociation is open reduction.^[4]^ Therefore, the surgeon should consider possible modification of any prosthesis components or convert it to total hip arthroplasty. In addition, various factors such as soft tissue tension and muscle weakness should be considered. In the present case, hemiplegia was caused by a brain tumor, and muscle weakness was severe. A total of 3 dislocations occurred, and we believe that it was caused by a growing brain tumor. Although the posterolateral approach was performed and the integrity of the short external rotator repair could not be completely ruled out, the surgery was performed normally, and the first dislocation occurred 8 weeks following surgery. Therefore, the degree of the muscle weakness became more severe because of the enlargement of the brain tumor. During the revision surgery, a soft tissue tension was considered by adjusting the neck length in the same dual locking implant, and the prosthesis was confirmed to be stable throughout its full range of motion. Although the follow-up period was short, no further dislocations occurred until his death.

Although the number of reports is small and it is difficult to generalize, we believe that our case report suggests a lesson to be careful when performing closed reduction of bipolar dislocation. In this case, hemiplegia was caused by a brain tumor, and the effect of muscle atrophy cannot be ruled out. Also, we cannot assure that bipolar cup dissociation may occur even in the case of the “O” sign we suggested. Therefore, it is considered that verification through application in more cases is necessary. Early bipolar dissociation is an iatrogenic complication that can occur during manual reduction of the bipolar dislocation and requires certain precautions. Thus, closed reduction of bipolar dislocation should be attempted very cautiously, paying attention to the shape of the cup and head component while observing fluoroscopic images under sufficient sedation. In addition, application of any kind of torsional forces over the posterior acetabular rim should be avoided.

## Author contributions

**Data curation:** Sang-Woo Son.

**Writing – original draft:** Seung-Bo Sim.

**Writing – review & editing:** Bum-Jin Shim.
